# Input and benchmarking data for flow simulations in discrete fracture networks

**DOI:** 10.1016/j.dib.2018.10.088

**Published:** 2018-10-26

**Authors:** Alessio Fumagalli, Eirik Keilegavlen, Stefano Scialò

**Affiliations:** a Department of Mathematics, University of Bergen, Allègaten 41, Bergen 5007, Norway; b Dipartimento di Scienze Matematiche, Politecnico di Torino, Corso Duca degli Abruzzi 24, 10129 Torino, Italy; c INdAM Research Group GNCS, Italy

## Abstract

This article reports and describes the data related to the paper “Conforming, non-conforming and non-matching discretization couplings in discrete fracture network simulations” (Fumagalli et al., 2019). The data provided include a set of geometrical input data of Discrete Fracture Networks (DFNs) and a set of simulation results. The geometrical data describe the geometry of fracture networks of increasing complexity. These data also include the geometry of a DFN extruded from a real fracture outcrop in Western Norway. Simulation results are obtained using several different numerical schemes and provide convergence history, plots over line and upscaled output quantities related to the various considered geometries.

**Specifications table**

**Table:** 

Subject area	Mathematics
More specific subject area	Numerical Analysis, Fractured Porous Media
Type of data	Tables,.csv files
How data was acquired	Artificial test cases, Elaboration of geological measurements, Computer simulations
Data format	Raw
Experimental factors	Simulations are based on the Discrete Fracture Network model for subsoil description.
Experimental features	Conforming, partially conforming and non-matching meshes are used, and seven different numerical schemes.
Data source location	Algerøyna, Bergen Municipality, Western Norway - GPS coordinates: 60°20′19.8′′N and 4°55′54.7′′E (applicable to part of the data)
Data accessibility	Data is available with this article
Related research article	A. Fumagalli, E. Keilegavlen, S. Scialò, Conforming, non-conforming and non-matching discretization couplings in discrete fracture network simulations, [1]

**Value of the data**

• Data include several geometrical input data for Discrete Fracture Network simulations, useful to reproduce and benchmark numerical experiments.
• Data on a fracture network extruded from a real outcrop in Western Norway is provided, useful for validation tests on realistic networks.
• Simulation results relative to a broad range of numerical schemes is provided to allow for benchmarking and comparisons.

## 1 Data

Data provided in the present article is related to numerical experiments in Discrete Fracture Networks (DFNs), and can be divided into two macro categories: i) geometrical input data of the networks, and ii) simulation results of seven different numerical schemes applied to the various geometries.

DFNs are set of intersecting polygons resembling networks of fractures in the subsoil and are complex geometrical domains [2]. A variety of numerical schemes is available to perform simulations in such domains, which, for the purpose of this presentation, can be divided into three categories, according to the kind of mesh that can be used for the simulations:

• conforming to the intersections between polygons,
• partially conforming to the geometry, i.e. allowing for hanging nodes,
• non-matching, allowing mesh elements to arbitrarily cross the intersections among the fracture polygons.

## 2 Experimental design, materials, and methods

Here data resulting from seven approaches using conforming, partially conforming and non-matching meshes are included. These methods are synthetically described in Table 1. More details can be found in Ref. [1] and in the references. Conforming triangular meshes are produced using Gmsh [9], non-matching triangular meshes are generated with Triangle [10], whereas the results are partly produced with the PorePy library [8], available at http://github.com/pmgbergen/porepy.

**Table 1:** Summary of numerical methods. (*): only for Setting 5.

Method description and references	Label	Grid type
Optimization and XFEM [3]	OPT-XFEM	Non-matching
Optimization and FEM [3]	OPT-FEM	Non-matching
VEM conforming [4]	VEM-C	Polygonal conforming
VEM Mortar [4]	VEM-M	Polygonal non-conforming
Mixed VEM conforming [5]	MVEM	Polygonal conforming
Mixed VEM conforming [6], [8]	MVEM-CONF	Triangular conforming/non-conforming(*)
Mixed VEM coarsening [6], [8]	MVEM-COARSE	Polygonal conforming/non-conforming(*)
Multi Point Flux Approximation [7], [8]	MPFA	Triangular conforming/non-conforming(*)
Two-Point Flux Approximation [8]	TPFA	Triangular conforming/non-conforming(*)

The data included in the present work refer to five different settings, all including both geometrical data and simulation results. The general structure of the dataset is the following. The data of each setting is contained in a different folder. Each folder contains:

• Geometrical data, either as a GEOM.dfn file or as a Matlab script generating several GEOM_j.dfn file, where *j* is an integer differentiating the various files.
• The Matlab script make_plot.m post processing the simulation result data
• A sub-folder data, containing the raw simulation results. Simulation result files are csv files with filenames having the following structure: *MethodLabel*_*DataType*_*Grid*.csv, being *MethodLabel* one of the labels in Table 1 identifying the method, *DataType* a string identifying the kind of data contained in the file, as detailed in the following, and *Grid* an identifier of the size of the mesh, larger for finer meshes. Please note that for files containing data on multiple grids the field *Grid* is empty. Four *DataType* specifiers are present: “*convergence*”, “*p_o_l_j*”, “*fluxes*”, “*cells*”.

***The “GEOM.dfn” files***. The GEOM.dfn file has the following structure. The first line contains the keyword FRACTURES followed by an integer “k” specifying the number of fracture-resembling polygons in the file. Then, for each polygon, the following lines specify: the fracture id (an integer between 0 and *k*−1), an integer *np* setting the number of vertexes of the fracture polygon, and then *np* lines each containing the vertex id (an integer between 0 and *np−1*) followed by three real numbers in double precision for the 3D coordinates of the vertex.

FRACTURES k.

0 np.

0 Vx Vy Vz.

….

np-1 Vx Vy Vz.

***The “convergence” files.*** These files contain data on the error norms of the numerical schemes when the size of the computational mesh is reduced.

***The “p_o_l_j” files.*** These files contain plots of the computed solution over specific lines against arc length. The index *j* refers to the specific geometry reported in the file GEOM_j.dfn.

***The “fluxes” files.*** These files contain data on the overall flux flowing through the network of fractures for different geometries.

***The “cells” files.*** These files contain data on the number of mesh elements produced by the various methods for different geometries.

***Setting 1.*** The first setting consists of a network of three orthogonal fractures. Data related to this setting are collected in the compressed folder *test1*. This setting contains a single geometry, and convergence data are provided for the various methods against a known analytical solution, also provided as a Matlab function. Scripts for data post-processing are given.

***Setting 2.*** The second setting consists of a network of three fractures. Data related to this setting are collected in the compressed folder *test2.* The geometry in this case is variable, as the angle between two of these fractures is allowed to change. The script “generate_geometries.m” produces 20 geometry files GEOM_*j*.dfn, j=1,…,20, as the variable angle between the two fractures varies between π/2 and π/565. Plot over line files are provided over a segment γ located at x=0.35,z=0 as y varies between 0 and 1 (reference system as the one in the geometry files), for the geometries *j* = 1 and *j* = 20. Flux and cells files are also given.

***Setting 3.*** Also, this setting consists of a network of three fractures. Data related to this setting are collected in the compressed folder *test3.* The geometry is variable, as two of the three fractures move towards disconnection from the network, thus generating an intersection of vanishing length. The script “generate_geometries.m” produces 21 geometry files GEOM_*j*.dfn, j=1,…,21, as the length of the intersection line varies between 0.6 and 0.01. Plot over line files are provided over a segment γ located at x=0.5,y=0.5 as z varies between *z*_min_ and *z*_max_ of the network (reference system as the one in the geometry files), for the geometries *j* = 1 and *j =* 20. Please observe that the line γ is located on one of the fractures that changes position in this setting, and thus also its position changes. Flux files are given.

***Setting 4.*** This setting consists of a network of 10 fractures. Data related to this setting are collected in the compressed folder *test4*. The geometry is variable, as one of the fractures in the network reduces its length. The script “generate_geometries.m” produces 44 geometry files GEOM_*j*.dfn, j=1,…,44, as the length of the fracture varies between 2.0 and 0.26. Plot over line files are provided over a segment γ located at x=1.5,z=0.5 as y varies between 0 and 2 (reference system as the one in the geometry files), for the geometries *j* = 1 and *j* = 44. Flux files are given.

***Setting 5.*** This setting consists of a network of 89 fractures, extruded from a real outcrop located in Western Norway at GPS coordinates 60°20′19.8′′N and 4°55′54.7′′E. From the 2D data, a 3D network is extruded by creating circular discs (by randomly relating the disc center to the outcrop) which are consistent with the outcrop lines. Care is taken to preserve the intersection type of fractures – if two fractures meet in a T-intersection in 2D, this is preserved for the extruded fractures.

Data related to this setting are collected in the compressed folder *test5.* The geometry is fixed and described by the GEOM.dfn file. Output data files are contained in the “data” sub-folder of the *test5* folder and differ from the output files of the previous setting. Data is collected in *MethodLabel*_*direction.txt* files, where “*direction*” is one among: *“bottom_top”*, “*back_front*”, “*left_right*”, corresponding to the *x*, *y*, and *z* direction, with respect to the coordinate system of the geometry file. The “*MethodLabel*” field is one of the labels in Table 1, with the optional suffix “-star”, denoting the non-conforming version of the method. The data reported in these files represent the flux flowing through the network when a unitary pressure drop is imposed along the corresponding direction.
