# Supplementary material for: Input and benchmarking data for flow simulations in discrete fracture networks
Source: Data Brief. 2018 Oct 26;21:1135–9. doi: 10.1016/j.dib.2018.10.088 (PMC6231035; doi:10.1016/j.dib.2018.10.088)
Supplement: Supplementary file 1 — Transparency document. [file mmc1.pdf]

Declarations of interest: none

Best regards,

Alessio Fumagalli, Eirik Keilegavlen, Stefano Scialò
